# Impact of RSV test positivity, patient characteristics, and treatment characteristics on the cost of hospitalization for acute bronchiolitis in a French university medical center (2010–2015)

**DOI:** 10.3389/fped.2023.1126229

**Published:** 2023-07-14

**Authors:** Benoit Dervaux, Marine Van Berleere, Xavier Lenne, Marine Wyckaert, François Dubos

**Affiliations:** ^1^ CHU Lille, Direction de la Recherche et de l’Innovation, Lille, France; ^2^ CHU Lille, Département d’Information Médicale, Lille, France; ^3^ CHU Lille, Urgences pédiatriques & maladies infectieuses, Lille, France; ^4^ Univ Lille, ULR 2694 - Évaluation des Technologies de Santé et des Pratiques Médicales, Lille, France

**Keywords:** bronchiolitis, cost analysis, hospitalization costs, RSV infections, viral respiratory infection

## Abstract

**Background:**

In young children, respiratory syncytial virus (RSV)-related bronchiolitis is typically more severe than other respiratory tract infections, with a greater need for oxygen therapy and respiratory support. Few studies have compared the cost of hospitalization with regard to virological status. The objective of this study was to compare the costs of hospitalization for RSV-positive vs. RSV-negative bronchiolitis in a French university medical center between 2010 and 2015.

**Methods:**

The cost models were compared using conventional goodness-of-fit criteria. Covariates included the characteristics of the patients, pre-existing respiratory and non-respiratory comorbidities, superinfections, medical care provided, and the length of stay.

**Results:**

RSV was detected in 679 (58.3%) of the 1,164 hospital stays by children under 2 years with virological data. Oxygen therapy and respiratory support were twice as frequent for the RSV-positive cases. The median hospitalization cost was estimated at €3,248.4 (interquartile range: €2,572.1). The cost distribution was positively skewed with a variation coefficient (CV = standard deviation/mean) greater than one (mean = €4,212.9, standard deviation = €5,047, CV = 1.2). In univariate analyses, there was no significant cost difference between the RSV-positive and RSV-negative cases. In the best multivariate model, the significant positive effect of RSV positivity on cost waned after the introduction of medical care variables and the length of stay. The results were sensitive to the specification of the model.

**Conclusions:**

It was impossible to firmly conclude that hospitalization costs were higher for the RSV-positive cases.

## Introduction

Acute bronchiolitis is the most frequent acute lower respiratory tract infection in infants (1) and constitutes the primary indication for hospital admission in children under the age of 12 months (2). Respiratory syncytial virus (RSV) is the leading cause of acute lower respiratory tract infections in children worldwide (3). Acute RSV-related infections place a heavy burden on healthcare systems (4, 5). Better knowledge of the hospitalization costs related to acute bronchiolitis is a prerequisite for cost-effectiveness analyses of innovative vaccines, antivirals, and prophylactic treatments against RSV-related infections (6).

The impact of the viral etiology on the course of respiratory disease has been described in the literature. Several studies have shown that RSV-related infections are more severe than infections caused by other respiratory tract viruses (7–14). In contrast, a number of studies concluded that RSV was not a severity factor (15, 16), and others were inconclusive (17, 18). Apart from methodological differences, interstudy differences in the study population might explain these discrepant findings. One can reasonably suggest that the characteristics of the patients on admission (age, underlying medical conditions, etc.) are linked to the hospitalization cost independently of the viral etiology. In most studies, the RSV-positive patients were younger than the RSV-negative patients, whereas the latter had a greater comorbidity burden on admission. As the length of stay (LOS) and admission to pediatric intensive care units (PICUs) were known to be the most important cost drivers in hospitals, a more severe course of disease was expected to generate higher costs. Surprisingly, only two studies had analyzed hospitalization costs with regard to the viral etiology by comparing costs for the RSV-positive vs. RSV-negative cases: one found higher costs for the RSV-positive patients (13), and the other found that the difference was not significant (15). Lastly, a Japanese study of children with respiratory tract infections found that hospitalization costs were lower when RSV was the primary etiology (4).

Hospitalization cost distributions are generally highly skewed and heavily tailed; in order to handle these distributional characteristics, a number of models have been developed and described in the literature (19). After a comparison of the various models, the most appropriate one can be selected.

The objectives of the present study were to estimate the cost of hospitalization for acute bronchiolitis in children under 2 years of age and to compare resource use in the RSV-positive vs. RSV-negative cases, after controlling for a wide range of covariates.

## Material and methods

### Study design

We carried out a retrospective, observational study of routine healthcare data collected at Lille University Medical Center (Lille, France) between 2010 and 2015. The present report complied with the Strengthening the Reporting of Observational Studies in Epidemiology (STROBE) statement. We included all children under 2 years of age and who were admitted to hospital through the pediatric emergency department (PED) with a diagnosis of bronchiolitis. In our experience, almost all the children admitted to hospital for bronchiolitis first attend the PED. The statistical unit was the hospital admission, given that few children were admitted more than once during the 6-year study period. Multiple admissions were considered as independent observations.

### Inclusion and exclusion criteria

We included all the children admitted for acute bronchiolitis through the PED. The diagnosis recorded in de-identified electronic medical records was confirmed by combining the International Classification of Diseases, 10th Revision (ICD-10) code and a compatible diagnosis-related group (DRG) code. The following inclusion and exclusion criteria were applied to selected cases: age under 24 months on admission, admission through the PED between 1 January 2010 and 31 December 2015, an entire stay at Lille University Medical Center (i.e., no transfers to or from another hospital) and an ICD-10 code for acute bronchiolitis due to RSV (J21.0), human metapneumovirus (J21.1), other specified organisms (J21.8), or unspecified organisms (J21.9). The following DRG codes were applied: 04M02 “Bronchitis and asthma, age under 18”; 04M04 “Simple pneumonia and pleurisy, age under 18”; 04M06 “Respiratory infections and inflammation, age under 18”; 04M18 “Bronchiolitis”; and 04M13 “Pulmonary edema and respiratory distress,” irrespective of the level of severity. Cases were excluded if virological test data were missing.

### Statistical power and sample size

During the study period, approximatively 2,000 children below the age of 24 months were admitted through the PED to Lille University Medical Center for acute bronchiolitis. In view of this sample size, we assessed the difference in mean cost between the RSV-positive cases and RSV-negative cases that could be detected for a predefined statistical power. Two data sets were used to estimate the mean [standard deviation (SD)] costs: (i) using the French national hospital discharge database (20), we counted the number of hospital stays with the selected ICD-10 codes over a 3-year period (2013–2015) in French university medical centers; and (ii) the mean cost per DRG and the corresponding standard error of the mean were obtained from the French national cost scale for 2015 (Supplementary Table S1).

Assuming that the mean (SD) cost of a hospital stay was €2,403 (€5,335) (Supplementary Table S1) and considering a two-tailed comparison of means (alpha = 0.05, power = 0.80) and an RSV positivity rate of 60% (1, 21), it was possible to detect an intergroup cost difference (i.e., RSV-positive cases vs. RSV-negative cases) of €682 per hospital stay with a sample size of 2,000 and €788 with a sample size of 1,500. This potential difference was approximatively equivalent to the mean daily cost of a bed in a French tertiary hospital.

### Data collection

All included children were tested by nasopharyngeal swab sampling for a panel of respiratory viruses, using direct immunofluorescence antibody assays or multiplex PCR tests. In our center, the use of PCR has been steadily increasing since 2013. In 2015, direct immunofluorescence accounted for two-thirds of the tests, and multiplex PCRs accounted for the remaining third. Over the study period, the children were systematically screened for RSV but not always for the other viruses. The virological test results were analyzed with regard to RSV status. Positivity for other respiratory viruses was also noted. A comorbidity was defined as an underlying condition (according to the medical records of the patient) known to be a risk factor for severe bronchiolitis [i.e., premature birth (<37 weeks of amenorrhea), chronic respiratory failure, bronchopulmonary dysplasia, cystic fibrosis, lung or respiratory tract malformation, congenital cardiac defects causing hypoxemia, immunodeficiency disorders, Down’s syndrome, severe swallowing disorders, and neuromuscular disease] (22, 23). The data on the features and outcome of the respiratory infection [fever, the provision of oxygen therapy or respiratory support (including mechanical ventilation), radiographic pattern, and superinfections] were also collected. Lastly, LOS and admission to the PICU were reported.

### Variables

Most variables were encoded as binary variables. The virology results were encoded as “RSV-positive” or “positive for other viruses” (rhinovirus, metapneumovirus, myxovirus parainfluenzae, myxovirus influenza, adenovirus, bocavirus, and other viruses). Comorbidities were categorized as “respiratory comorbidities” if they could compromise respiratory function or as “other comorbidities” if not. Likewise, superinfections were categorized as “pulmonary” or “other” (gastrointestinal, ENT, skin, etc.). Age was measured in months and was categorized for analysis as a three-level variable (<2 months, from 2 to less than 6 months, and ≥6 months). The LOS was analyzed as a continuous variable on a log scale.

The costs of hospital stays included in this study were obtained from the analytical accounting system at Lille University Medical Center. In French hospitals, analytical accounting is managed in accordance with the national guidelines monitored by the French Ministry of Health. Few costs are directly attributable to hospital stays (e.g., drugs, implantable medical devices); most are measured at the ward level and then reallocated to hospital stays using allocation keys. The evaluation of costs depends on these accounting conventions. However, and with the exception of difficult-to-conduct bottom-up approaches (such as micro-costing), accounting cost is the best available measure of inpatient resource use. All costs were expressed in 2015 euros. Costs arising in the previous years were adjusted using the hospital care price index (Supplementary Table S2).

The data collection, processing, and storage were registered with the data protection officer of Lille University Medical Center (reference: DEC16–274) and complied with the French legislation. In line with the French legislation on retrospective analyses of de-identified data from routine clinical practice, authorization by an institutional review board was not required.

### Statistical analyses

#### Initial descriptive analyses

In univariate analyses, the RSV-positive cases were compared with RSV-negative cases in terms of all the other study variables. For categorical variables, we applied a chi-squared test or (when required) Fisher’s exact test. For quantitative variables other than cost, we applied the Student’s *t*-test (for normally distributed data) or the Kruskal–Wallis test (for other distributions). The mean costs were analyzed with regard to all the categorical variables, including the viral etiology. The differences between means were tested with a non-parametric bootstrap test. For all tests, the threshold for statistical significance was set to *p* < 0.05. The results were not corrected for multiple testing.

To test the influence of the viral etiology on the course of hospital stays, logistic models were built with variables describing the medical care of the patients (PICU admission, oxygen therapy, respiratory support, or x-ray imaging) as dependent variables and the characteristics of the patients on admission (age, sex, prematurity, and comorbidities) and the virological test result as independent covariates.

#### Cost models

a) Functional forms
  Several cost model specifications were compared: ordinary least squares (OLS) regressions with a transformed cost variable; generalized linear models (GLM) with various link functions and family distributions; hazard models; and semiparametric models.
  1. For OLS regressions, we first considered conventional log transformation and then ran Box-Cox regression models.
  2. Concerning GLMs, the extended estimating equations approach (EEE) (24) was used to select the adequate link function and family distribution simultaneously. Most of the usual GLMs with log link functions and gamma/inverse Gaussian family distributions were also considered as benchmarks.
  3. With regard to hazard models, a generalized gamma model was first estimated. Nested specifications of the generalized gamma model (lognormal, Weibull, standard gamma, and exponential) were then tested.
  4. Concerning semiparametric models, a discrete conditional density estimator was provided (25, 26). Data were ranked according to actual cost, the sample was subdivided into 10 strata (i.e., deciles), and the mean cost was computed within each stratum. The probabilities with which observations belonged to the predefined intervals were estimated using an ordered logit model (ORL) or a multinomial logit model (MNL). Predicted costs were computed as the vector product of probabilities and intervals of the mean cost.
b) Choice of independent variables
  Covariates were successively introduced. The virological test result—the main focus of our analysis—was always an independent variable. The characteristics of the patients on admission were introduced first (Model 1). The variables describing the medical care of the patients and the occurrence of adverse events during the hospital stay were then added (Model 2). Lastly, the LOS (on a log scale) was included in the independent variables as a proxy for unobserved severity (Model 3). Model 2 was considered to be the model of interest because LOS is not an independent predictor of cost but is an outcome *per se* and is at least partly endogenous.
c) Goodness-of-fit tests
  The following modeling strategy was implemented to compare models and select the most accurate one. First, goodness-of-fit tests (Pregibon’s link test, Pearson’s rho test, and the modified Hosmer–Lemeshow test) were applied, and the prediction errors [the mean absolute prediction error (MAPE), the mean prediction error (MPE), and the root mean square error (RMSE)] were checked. Overfitting was assessed using the Copas test, with the full sample randomly allocated to an estimation subsample (80%) and a forecast subsample (20%). When overfitting was rejected, the final model was re-estimated on the full sample. In a second step, the information criteria [the Akaike information criterion (AIC) and the Bayesian information criterion (BIC)] were considered. All testing procedures were conducted on Model 2 (the “full” model). The statistical analyses were performed using Stata software (version 15.0, StataCorp, College Station, TX, United States).

## Results

### Descriptive analyses

The virological test data were available for 1,164 (68%) of the 1,717 hospital stays meeting the inclusion criteria of the study. The cases lacking virological test data had a shorter LOS, were classified in less severe DRGs, and concerned older children (Supplementary Table S3). When hospital stays of less than three nights were excluded, the proportion with virology testing data was 82%.

The virology test was informative in 804 cases (69.1%) (Table 1). RSV was detected in 679 (58.3%) cases, i.e., 84% of the cases with informative virological test data. Of the RSV-positive cases, 4.6% were also positive for one or more other viruses. A total of 188 cases (16.2%) had comorbidities, and there was one in-hospital death (an RSV-positive case). The RSV-negative infants were older and more likely to have been born prematurely and had more comorbidities. Infections with other viruses were more frequent among the RSV-negative cases. Oxygen therapy was more frequent among the RSV-positive cases.

**Table 1:** Descriptive statistics for RSV status (full sample, *N* = 1,164).

Variables	RSV-negative	RSV-positive	All	*p*-values for RSV-negative vs. RSV-positive
	(*n* = 485, 41.7%)	(*n* = 679, 58.3%)	(*n* = 1,164)	
Positive for other respiratory viruses (%)	125 (25.8)	31 (4.6)	156 (13.4)	0.000
Age: median (months) (IQR)	3.0 (2.0–7.0)	2.0 (1.0–5.0)	3.0 (1.0–6.0)	
Sex (male sex = 1) (%)	296 (61.0)	353 (52.0)	649 (55.8)	0.002
Preterm (%)	123 (25.4)	125 (18.4)	248 (21.3)	0.004
Respiratory comorbidities (%)	55 (11.3)	36 (5.3)	91 (7.8)	0.000
Other comorbidities (%)	67 (13.8)	55 (8.1)	122 (10.5)	0.002
Length of stay: median (nights) (IQR)	4.0 (3.0–7.0)	5.0 (3.0–8.0)	5.0 (3.0–7.0)	
PICU admission (%)	41 (8.5)	51 (7.5)	92 (7.9)	0.56
Fever (%)	197 (40.6)	319 (47.0)	516 (44.3)	0.0
Oxygen therapy (%)	314 (64.7)	539 (79.4)	853 (73.3)	0.000
Respiratory support (%)	45 (9.3)	85 (12.5)	130 (11.2)	0.08
X-ray imaging (%)	358 (73.8)	494 (72.8)	852 (73.2)	0.69
Pulmonary superinfection (%)	73 (15.1)	115 (16.9)	188 (16.2)	0.39
Other superinfection (%)	51 (10.5)	61 (9.0)	112 (9.6)	0.38
Cost: mean (€_2015_) ± SD	4,282.5 ± 6,166.4	4,163.1 ± 4,068.1	4,212.9 ± 5,047.3	0.002
Cost: median (€_2015_) (IQR)	2,902.3 (2,773.7)	3,393.6 (2,314.9)	3,248.4 (2,572.1)	

There was no difference between the RSV-negative and RSV-positive cases regarding the mean LOS (whereas the median LOS was longer for the RSV-positive cases), PICU admission, x-ray imaging, and the occurrence of a superinfection.

After controlling for the characteristics of the patients, the odds ratio associated with the “RSV-positive” variable was 2.05 (95% CI = 1.52‒2.79) for oxygen therapy and 1.64 (95% CI = 1.02‒2.65) for respiratory support. The odds ratio did not significantly differ from unity for x-ray imaging and for PICU admission. The “positive for another virus” variable was never statistically significant (Supplementary Table S4).

### Univariate analyses of cost

The cost distribution of the sample was highly skewed and heavily tailed (Supplementary Table S4). Although log transformation reduced the skewness, the level of kurtosis remained high. In univariate comparisons, the RSV-positive and RSV-negative cases did not differ with regard to the mean cost (whereas the median cost was higher for the RSV-positive cases). The mean cost did not depend on the sex. Positivity for other viruses, prematurity, other comorbidities, and the occurrence of superinfection were associated with a higher cost. The variables describing the medical care received for respiratory failure and admission to the PICU increased the cost of hospitalization by a factor of between 2 and 3 (Table 2).

**Table 2:** Comparisons of the mean cost in a univariate analysis (full sample, *N* = 1,164).

Variables	No/without	Yes/with	Bootstrapped
	mean ± SD cost	mean ± SD cost	95% CI^a^
RSV-positive	4,282.5 ± 6,166.4	4,163.1 ± 4,068.1	−782.1 to 437.8
Positive for other respiratory viruses	4,003.7 ± 4,664.9	5,564.2 ± 6,902.7	570.1 to 2,773.7
Sex (male sex = 1)	4,508.8 ± 6,153.5	3,978.1 ± 3,944.9	−1,208.7 to 12.0
Preterm	3,786.9 ± 2,988.1	5,786.0 ± 9,149.7	954.7 to 3,258.1
Respiratory comorbidities	3,996.8 ± 3,836.7	6,761.0 ± 12,113.8	726.3 to 5,726.1
Other comorbidities	3,937.9 ± 3,638.6	6,561.6 ± 11,168.8	945.8 to 5,081.3
Fever	4,147.5 ± 5,558.4	4,295.0 ± 4,324.7	−400.3 to 723.3
Pulmonary superinfections	3,866.2 ± 4,643.5	6,012.8 ± 6,490.8	1,219.4 to 3,047.6
Other superinfections	4,019.6 ± 4,816.9	6,027.9 ± 6,599.0	763.4 to 3,433.4
PICU admission	3,764.9 ± 4,230.4	9,432.8 ± 9,221.8	3,859.1 to 7,573.6
Oxygen therapy	2,549.0 ± 2,128.1	4,819.5 ± 5,634.5	1,845.5 to 2,755.3
X-ray imaging	2,986.1 ± 2,101.5	4,662.1 ± 5,696.3	1,276.5 to 2,169.0
Respiratory support	3,415.2 ± 2,098.6	10,557.3 ± 12,196.0	5,184.1 to 9,514.3

^a^ Bootstrapped credibility interval for the difference in means (yes/with vs. no/without)—percentile method.

### Multivariate analyses

a) Selection of the most accurate model

Concerning the OLS regressions, heteroscedasticity was detected on the log-transformed cost. The maximum likelihood estimator for the Box-Cox transformation coefficient was −0.11 (95% CI = −0.14 to −0.07). No further heteroscedasticity was further detected. Concerning GLMs, the distribution of log-scale errors for the log-gamma GLM was nearly symmetric (skewness = 0.04, *p* = 0.65) but was heavily tailed (kurtosis = 3.54, *p* = 0.004). A modified Park’s test rejected both the gamma and inverse Gaussian families. The EEE approach identified an inverse square root link function (estimated Box-Cox transformation coefficient = −0.38, 95% CI = −0.72 to −0.05) and a family between gamma and inverse Gaussian (estimated power coefficient = 2.58, 95% CI = 2.10–3.05) (Supplementary Table S5). Concerning the hazard models, the kappa coefficient was not significantly different from zero for the generalized gamma, (*κ *= −0.06, 95% CI = −0.22 to 0.10), which suggested a heteroscedastic lognormal distribution for cost (Supplementary Table S6).

**Figure 1:**
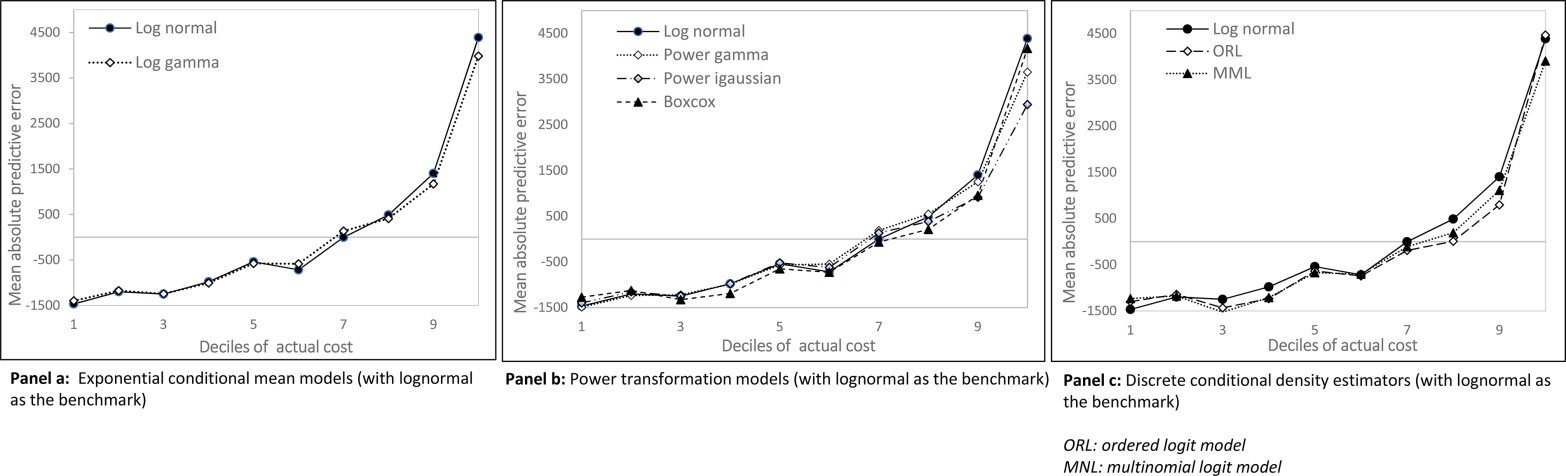
Mean prediction error per decile of actual cost (forecast subsample, *N* = 233).

The results of specification tests and goodness-of-fit criteria are summarized in Supplementary Tables S7, S8. The Pearson’s rho test and the modified Hosmer–Lemeshow test were the most critical tests. For all model specifications, the correlation between raw residuals and fitted values was significant, indicating a degree of misspecification. The correlation was lowest for the log-gamma GLM and the lognormal model. Discrete conditional density estimators and the Box-Cox model failed the modified Hosmer–Lemeshow test. The Copas test did not detect any overfitting. The estimation subsample (*n* = 931) and the forecast subsample (*n* = 233) did not significantly differ with regard to the proportion of the RSV-positive cases (57.6% vs. 61.4%, respectively) or the mean cost (€4,225.90 vs. €4,160.70, respectively). The MAPE was lowest for the lognormal model and the Box-Cox model, although the log-gamma GLM had the lowest RMSE. In the forecast subsample, the lognormal model yielded the smallest MPE, MAPE, and RMSE. In the estimation subsample, the AIC and BIC were lowest for the lognormal model and the Box-Cox model. However, all the models had very similar predictive abilities (Figure 1). All the models overestimated the actual cost in the first seven deciles and underestimated the actual cost at the tail of the distribution. Ultimately, two models were selected: the lognormal model and the log-gamma GLM.

b) The main cost drivers

The lognormal model (Table 3) and the log-gamma GLM (Table 4) gave similar results. The cost was lower for males than for females and was significantly higher for children below 2 months of age than for older children. Prematurity and pre-existing comorbidities were associated with a higher hospitalization cost, as were the occurrence of superinfections and medical care for respiratory failure. Here, RSV positivity did not have an impact on the hospitalization cost. Although the RSV-related infections increased the cost in the lognormal model when solely the characteristics of the patients were controlled for, the relationship weakened when other explanatory variables were included in the model. In the standard log-gamma GLM model, the RSV-related infections did not affect the hospitalization cost—even when considering the most parsimonious model. The detection of other respiratory viruses always had a significant, positive (increasing) impact on cost.

**Table 3:** The results for the lognormal model (full sample, *N* = 1,164).

Variables	Model 1		Model 2		Model 3	
	OR	95% CI	OR	95% CI	OR	95% CI
RSV-positive	1.17**	1.08 to 1.27	1.06***	0.99 to 1.13	1.00	0.98 to 1.03
Positive for other viruses	1.18*	1.01 to 1.38	1.20**	1.06 to 1.35	0.98	0.94 to 1.02
RSV-positive × positive for other viruses	1.16	0.84 to 1.60	1.13	0.91 to 1.40	1.00	0.92 to 1.08
Sex (male sex = 1)	0.91**	0.85 to 0.97	0.91**	0.87 to 0.96	0.98*	0.96 to 1.00
Age <2 months	1.22**	1.11 to 1.34	1.24**	1.14 to 1.34	0.78**	0.75 to 0.81
Age 2–6 months	1.00	0.92 to 1.09	1.04	0.97 to 1.12	0.95**	0.92 to 0.98
Age ≥6 months	Ref.	-	Ref.	-	Ref.	-
Preterm	1.21**	1.09 to 1.34	1.06*	1.01 to 1.17	0.97***	0.94 to 1.00
Respiratory comorbidities	1.19*	1.01 to 1.41	1.14*	1.01 to 1.30	1.02	0.97 to 1.06
Other comorbidities	1.30**	1.12 to 1.51	1.31**	1.16 to 1.48	1.09**	1.05 to 1.13
Pulmonary superinfections			1.18**	1.09 to 1.28	1.02	0.99 to 1.05
Other superinfections			1.26**	1.13 to 1.42	1.02	0.98 to 1.06
Oxygen therapy			1.61**	1.5 to 1.73	1.06**	1.03 to 1.09
X-ray imaging			1.13**	1.06 to 1.21	1.06**	1.03 to 1.09
Respiratory support			1.76**	1.57 to 1.98	1.25**	1.18 to 1.32
PICU admission			1.14*	1.00 to 1.30	1.14**	1.06 to 1.22
Ln(LOS)					2.83**	2.75 to 2.92
Constant	2,643.48		1,588.67		501.37	

OR, odds ratio.

* *p*-value ≤ 5%.

** *p*-value ≤ 1%.

*** *p*-value ≤ 10%.

ln(*σ*) was modeled as a linear function of independent variables. The “fever” variable was not included further in the statistical analyses because it had no impact on cost and its prevalence was similar for the RSV-positive cases and RSV-negative cases.

**Table 4:** The results for the log-gamma GLM (full sample, *N* = 1,164).

Variables	Model 1		Model 2		Model 3	
	OR	95% CI	OR	95% CI	OR	95% CI
RSV-positive	1.06	0.95 to 1.19	1.00	0.93 to 1.08	1.01	0.98 to 1.03
Positive for other viruses	1.23*	1.00 to 1.53	1.23**	1.06 to 1.43	0.98	0.94 to 1.02
RSV-positive × positive for other viruses	1.42***	0.94 to 2.15	1.14	0.86 to 1.49	1.01	0.93 to 1.10
Sex (male sex = 1)	0.89*	0.81 to 0.99	0.91**	0.85 to 0.97	0.98	0.96 to 1.01
Age <2 months	1.33**	1.18 to 1.50	1.27**	1.15 to 1.39	0.80**	0.78 to 0.83
Age 2–6 months	1.08	0.97 to 1.21	1.09*	1.00 to 1.19	0.96**	0.94 to 0.99
Age ≥6 months	Ref.	-	Ref.	-	Ref.	-
Preterm	1.36**	1.18 to 1.60	1.11*	1.02 to 1.22	0.98	0.95 to 1.01
Respiratory comorbidities	1.35*	1.05 to 1.74	1.21*	1.03 to 1.42	1.01	0.96 to 1.06
Other comorbidities	1.55**	1.27 to 1.89	1.50**	1.29 to 1.75	1.09**	1.05 to 1.13
Pulmonary superinfections			1.20**	1.10 to 1.32	1.04*	1.00 to 1.07
Other superinfections			1.39**	1.22 to 1.58	1.04***	1.00 to 1.08
Oxygen therapy			1.50**	1.38 to 1.63	1.06**	1.03 to 1.09
X-ray imaging			1.17**	1.08 to 1.26	1.08**	1.05 to 1.11
Respiratory support			2.09**	1.71 to 2.54	1.22**	1.15 to 1.28
PICU admission			1.12	0.93 to 1.35	1.18**	1.09 to 1.27
Ln(LOS)					2.82**	2.74 to 2.90
Constant	3,096.10		1,789.70		501.68	

OR, odds ratio.

** *p*-value ≤ 1%.

* *p*-value ≤ 5%.

*** *p*-value ≤ 10%.

The “fever” variable was not included further in the statistical analyses because it had no impact on cost and its prevalence was similar for the RSV-positive cases and RSV-negative cases.

The introduction of the LOS as an independent variable strongly impacted all the regression coefficients. The hospitalization cost was roughly proportional to the LOS. The medical care for respiratory failure (respiratory support and PICU admission, notably) still had positive impact on cost after controlling for the LOS. In this adjusted model, young age appeared to be a cost moderator: for a given LOS, the hospitalization costs were lower for children below 6 months of age than for older children, even though the mean LOS was higher in the younger age group. As expected, comorbidities and superinfection had positive coefficients. After adjustment for the LOS, the viral etiology had no impact on cost.

## Discussion

In our sample, the median hospitalization cost was estimated at €3,248.4 (interquartile range: €2,572.1); the estimated mean ± SD hospitalization cost for bronchiolitis was €4,212.9 ± €5,047. This was higher than the cost computed from all the DRGs related to bronchiolitis at the national level, including very short hospital stays (€2,403 ± €5,335). However, our estimates were consistent with a previous French study (27) and a recent systematic literature review (28). In the review, the estimated cost per episode of inpatient care without any follow-up was €4,712 on average and ranged from €1,530 in Europe to €6,315 in North America. Our results did not, however, demonstrate that hospitalization costs were higher for the RSV-related infections—even though the latter were associated with a higher incidence of respiratory support and oxygen therapy, both of which are proxy makers of disease severity.

A number of known risk factors increased the hospitalization cost. These variables were highly significant in all multivariate analyses, whatever the cost model specification considered. In univariate analyses, there was no significant mean cost difference between the RSV-positive and RSV-negative cases (€4,163 vs. €4,283, respectively), whereas the median cost was higher for the RSV-positive cases than for the RSV-negative cases (€3,393 vs. €2,902, respectively; *p* = 0.0018). Furthermore, the LOS was not significantly different (six nights, on average). In contrast, the cases for whom other respiratory viruses were detected had higher cost than the other cases (€5,564 vs. €4,004, respectively), with a significant longer LOS (2.7 nights longer, on average). The RSV-positive and RSV-negative cases appeared to have different sets of cost drivers. On one hand, respiratory support and oxygen therapy were more frequently prescribed for the RSV-positive cases. On the other, the RSV-negative cases were more frequently positive for other respiratory viruses, were more likely to be preterm, and had more comorbidities on admission. This observation might be explained (at least in part) by the fact that the at-risk children and those more likely to test positive for other viruses received palivizumab prophylaxis. Moreover, the proportion of children with chronic comorbidities is known to be high in our center (29). The impact of the RSV-related infections on cost varied from one model specification to another: it was significant and positive when considering the heteroscedastic lognormal model and non-significant when considering the gamma-log GLM. After the introduction of variables describing medical care, the coefficient for the “RSV-positive” variable became non-significant for all cost model specifications. After introducing the LOS as an independent variable, the viral etiology had no impact on cost; however, it must be borne in mind that LOS is highly correlated with cost and is so partly endogenous.

Our study had a number of strengths. First, our local guidelines promoted systematic virological testing during the study period for children admitted through the PED for bronchiolitis-related symptoms. Hence, the viral etiology was known for about two-thirds of hospital stays overall and 80% of stays of more than two nights. Second, the analytical accounting system in our medical center provided an estimate of the cost per hospital stay during the study. The study database therefore enabled us to evaluate how the inpatient cost for bronchiolitis varied as a function of the viral etiology. Third, we compared nine cost models in detail. According to the current international guidelines in this field, a variety of candidate models should be assessed with regard to their predictive ability; a particular model should not be selected *a priori* because the coefficients might vary from one specification to another.

The study also had some limitations. First, less severe cases were discharged rapidly, and virological test data were lacking for some of these, which generated selection bias. We characterized this bias for the available data: non-tested cases were less severe and had a lower cost than tested cases. Second, the study might be underpowered. For short hospital stays, the proportion of cases with virological test data was lower than expected (13.5%, for stays of less than three nights), which resulted in a smaller-than-expected sample size. In French tertiary hospitals, short stays are more frequent and account for 40% of the total. Considering the sample case-mix and the national reference cost per DRG in 2015, the mean estimated cost per case was €3,659 (Supplementary Table S3). However, the national reference cost is known not to be representative of tertiary hospitals (30). Third, the cost was measured imperfectly on the basis of the data from the analytical accounting system in our hospital; this was not the real economic cost reflecting the opportunity cost of the resources used. The opportunity cost is difficult to measure in healthcare systems to which market rules do not apply. Nevertheless, the 2020 guidelines of the French National Authority for Health on choosing methods for economic evaluation state that the cost obtained from a hospital’s analytical accounting system is the best available measure in the French setting. Fourth, the single-center design of the study means that the results might not be readily generalizable. The prevalence of complex cases is generally higher in a university medical center than in a general hospital. Fifth, some important explanatory variables might have been missing (e.g., severity scores or precise descriptions of symptoms on admission); this is a general limitation of retrospective studies. Sixthly, direct immunofluorescence testing was progressively replaced by multiplex PCR testing during the study period from 2010 to 2015. In most prospective studies, multiplex PCR is considered to be the gold standard. However, there was no reason to believe that this change in clinical practice influenced our comparison of the RSV-positive vs. RSV-negative cases. Our results must now be confirmed in a prospective, multicenter study of costs.

There are very few published data on the possible variation in bronchiolitis-related hospitalization costs as a function of the viral etiology. In studies of costs, these comparisons were at best ancillary analyses and were not conducted in depth. Our present study was designed to fill this knowledge gap. This is particularly important because a number of new monoclonal antibodies, vaccines, and anti-RSV drugs are at various stages in clinical development. Cost analyses do not have a financial perspective. In most European countries, marketing authorizations for prophylactic or therapeutic innovations depend on the availability of well-conducted, full-scale cost-effectiveness studies; cost analyses are essential inputs for the latter. Health economic evaluations and studies of the economic burden of disease based on retrospective hospital cost inputs are subject to the inherent limitations of these approaches, as described in detail here. In view of the associated uncertainties, the findings must be interpreted with caution.

## Data Availability

The raw data supporting the conclusions of this article will be made available by the authors, without undue reservation.

## RESCEU investigators

University of Antwerp: Philippe Beutels. University Medical Center Utrecht: Louis Bont. Servicio Galego de Saude: Federico Martinon-Torres. University of Turku and Turku University Hospital: Terho Heikkinen. University of Edinburgh: Harish Nair and Harry Campbell. University of Oxford: Andrew Pollard. Imperial College London: Peter Openshaw. National Institute for Public Health and the Environment: Adam Meijer. Statens Serum Institut: Thea K Fischer. University of Groningen: Maarten van den Berge. PENTA Foundation: Carlo Giaquinto. AstraZeneca: Michael Abram. Pfizer: Kena Swanson. GlaxoSmithKline: Bishoy Rizkalla. Sanofi Pasteur: Charlotte Vernhes and Scott Gallichan. Janssen: Jeroen Aerssens. Novavax: Veena Kumar. Team-IT Research: Eva Molero.
