# Supplementary material for: Impact of RSV test positivity, patient characteristics, and treatment characteristics on the cost of hospitalization for acute bronchiolitis in a French university medical center (2010–2015)
Source: Front Pediatr. 2023 Jul 14;11:1126229. doi: 10.3389/fped.2023.1126229 (PMC10390249; doi:10.3389/fped.2023.1126229)
Supplement: Supplementary file 1 [file Table1.docx]

**Supplementary Table 1.** The case mix at French university medical centers (2013-2015) and the mean (standard error) national reference cost per diagnosis-related group (DRG) (2015)

| DRG^a^ | Severity level | Number of hospital stays 2013-2015 | Mean cost (€) 2015 | Standard error (€) 2015 |
| --- | --- | --- | --- | --- |
| 04M02 | Short stay^b^ | 126 | 693.96 | 18.14 |
|  | Level 1 | 603 | 1723.37 | 60.75 |
|  | Level 2 | 368 | 2637.21 | 115.75 |
|  | Level 3 | 186 | 4629.31 | 249.48 |
|  | Level 4 | 0 | 11542.09 | 674.19 |
| 04M04 | Level 1 | 265 | 1533.13 | 43.32 |
|  | Level 2 | 312 | 3092.10 | 101.29 |
|  | Level 3 | 446 | 5338.30 | 304.35 |
|  | Level 4 | 0 | 19086.19 | 1153.07 |
| 04M06 | Short stay | 0 | 824.54 | 31.43 |
|  | Level 1 | 0 | 2969.11 | 177.72 |
|  | Level 2 | 12 | 5442.68 | 269.55 |
|  | Level 3 | 33 | 12450.48 | 806.65 |
|  | Level 4 | 0 | 15595.90 | 606.30 |
| 04M18 | Short stay | 19231 | 938.25 | 31.23 |
|  | Level 1 | 5893 | 2560.83 | 113.99 |
|  | Level 2 | 10004 | 2737.24 | 74.72 |
|  | Level 3 | 8976 | 4336.03 | 229.62 |
|  | Level 4 | 320 | 9497.64 | 702.96 |
| 04M13 | Short stay | 13 | 1252.71 | 232.47 |
|  | Level 1 | 624 | 1877.28 | 73.14 |
|  | Level 2 | 1135 | 5081.54 | 126.93 |
|  | Level 3 | 0 | 8408.60 | 216.34 |
|  | Level 4 | 0 | 22872.90 | 490.45 |

^a^ Most French DRGs have four levels of severity (level 1 to level 4). The level of severity is determined by a number of factors, including the presence of comorbidities, the patient's age, the length of stay, and the discharge destination. The prospective, DRG-based payments paid to French hospitals increasing with the level of severity.

^b^ Short or very short stays correspond to those in which the patient spends less than 3 nights in hospital; the patient is often discharged on the day of admission or on the following day.
