# Supplementary material for: Impact of RSV test positivity, patient characteristics, and treatment characteristics on the cost of hospitalization for acute bronchiolitis in a French university medical center (2010–2015)
Source: Front Pediatr. 2023 Jul 14;11:1126229. doi: 10.3389/fped.2023.1126229 (PMC10390249; doi:10.3389/fped.2023.1126229)
Supplement: Supplementary file 2 [file Table2.docx]

**Supplementary Table 2.** Hospital price index (public-sector hospitals, period 2010-2015)^[[1]](#footnote-1)^

|  | Price variation  Y/Y-1 (%) | Price index  2015=100 |
| --- | --- | --- |
| 2010 | -0.5 | 99.10 |
| 2011 | -0.2 | 98.90 |
| 2012 | 0.0 | 98.90 |
| 2013 | 0.8 | 99.70 |
| 2014 | 0.6 | 100.30 |
| 2015 | -0.3 | 100.00 |

1. https://drees.solidarites-sante.gouv.fr/sites/default/files/2020-07/cns2019.pdf [↑](#footnote-ref-1)
