# Supplementary material for: Impact of RSV test positivity, patient characteristics, and treatment characteristics on the cost of hospitalization for acute bronchiolitis in a French university medical center (2010–2015)
Source: Front Pediatr. 2023 Jul 14;11:1126229. doi: 10.3389/fped.2023.1126229 (PMC10390249; doi:10.3389/fped.2023.1126229)
Supplement: Supplementary file 3 [file Table3.docx]

**Supplementary Table 3.** Comparison of hospital stays with vs. without virological test data

| Variables | No test data available (N=553)  n (%) | Test data available (N=1164)  n (%) |
| --- | --- | --- |
| Diagnosis-related group (DRG) code: |  |  |
| “Bronchitis and asthma, age under 18” | 18 (3.3) | 27 (2.3) |
| “Simple pneumonia and pleurisy, age under 18” | 18 (3.3) | 49 (4.2) |
| “Respiratory infections and inflammation, age under 18” | 1 (0.2) | 6 (0.5) |
| “Bronchiolitis” | 364 (65.8) | 899 (77.2) |
| “Pulmonary edema and respiratory distress” | 152 (27.5) | 183 (15.7) |
|  |  |  |
| Level of severity: |  |  |
| Short stay | 262 (47.4) | 157 (13.5) |
| Level 1 | 153 (27.7) | 292 (25.1) |
| Level 2 | 72 (13.0) | 238 (20.4) |
| Level 3 | 56 (10.1) | 427 (36.7) |
| Level 4 | 10 (1.8) | 50 (4.3) |
|  |  |  |
| Age (mean ± SD) (months) | 6.2 ± 5.2 | 4.4 ± 4.3 |
| LOS (mean ± SD) (nights) | 2.9 ± 4.6 | 6.0 ± 5.4 |
|  |  |  |
| Cost (mean)^a^ (€) | 2271.2 | 3658.7 |

^a^: using the national reference cost per DRG for 2015

SD, standard deviation; LOS, length of stay
