# Supplementary material for: Impact of RSV test positivity, patient characteristics, and treatment characteristics on the cost of hospitalization for acute bronchiolitis in a French university medical center (2010–2015)
Source: Front Pediatr. 2023 Jul 14;11:1126229. doi: 10.3389/fped.2023.1126229 (PMC10390249; doi:10.3389/fped.2023.1126229)
Supplement: Supplementary file 4 [file Table4.docx]

**Supplementary Table 4.** Multivariate logistic models for PICU admission, oxygen therapy, respiratory support and X-Ray, after controlling for the patients’ characteristics

**PICU admission**

| **Variables** | **Odds ratio** | | **[95%CI]** |  |
| --- | --- | --- | --- | --- |
| RSV-positive | 1.02 |  | [0.59; 1.74] | |
| Positive for other viruses | 1.42 |  | [0.70; 2.88] | |
| RSV-positive x positive for other viruses | 0.49 |  | [0.09; 2.66] | |
| Sex (male sex = 1) | 0.85 |  | [0.54; 1.33] | |
| Age <2 months | 9.80 | ** | [4.50; 21.36] | |
| Age 2-6 months | 2.06 | ^#^ | [0.96; 4.43] | |
| Age ≥6 months | Ref |  | - | |
| Preterm | 3.23 | ** | [1.89; 5.50] | |
| Respiratory comorbidities | 1.78 |  | [0.80; 3.95] | |
| Other comorbidities | 2.43 | ** | [1.25; 4.73] | |
| Constant | 0.02 |  |  | |

PICU, Pediatric intensive care unit

**Oxygen therapy**

| **Variables** | **Odds ratio** | | **[95%CI]** |
| --- | --- | --- | --- |
| RSV-positive | 2.05 | ** | [1.52; 2.79] |
| Positive for other viruses | 0.71 |  | [0.47; 1.09] |
| RSV-positive x positive for other viruses | 0.85 |  | [0.34; 2.12] |
| Sex (male sex = 1) | 0.81 |  | [0.61; 1.07] |
| Age <2 months | 0.71 | ^#^ | [0.48; 1.05] |
| Age 2-6 months | 0.54 | ** | [0.38; 0.77] |
| Age ≥6 months | Ref |  | - |
| Preterm | 1.53 | * | [1.07; 2.21] |
| Respiratory comorbidities | 1.10 |  | [0.66; 1.93] |
| Other comorbidities | 0.65 | * | [0.42; 1.00] |
| Constant | 3.11 |  |  |

**Respiratory support**

| **Variables** | **Odds ratio** | | **[95%CI]** |
| --- | --- | --- | --- |
| RSV-positive | 1.64 | * | [1.02; 2.65] |
| Positive for other viruses | 1.41 |  | [0.72; 2.75] |
| RSV-positive x positive for other viruses | 2.31 |  | [0.79; 6.79] |
| Sex (male sex = 1) | 1.14 |  | [0.77; 1.69] |
| Age <2 months | 4.05 | ** | [2.17; 7.54] |
| Age 2-6 months | 2.80 | ** | [1.57; 5.00] |
| Age ≥6 months | Ref | Ref | - |
| Preterm | 2.53 | ** | [1.64; 3.91] |
| Respiratory comorbidities | 2.42 | ** | [1.30; 4.51] |
| Other comorbidities | 1.47 |  | [0.80; 2.71] |
| Constant | 0.02 |  |  |

**X-Ray**

| **Variables** | **Odds ratio** | | **[95%CI]** |
| --- | --- | --- | --- |
| RSV-positive | 1.01 |  | [0.74; 1.38] |
| Positive for other viruses | 0.96 |  | [0.60; 1.53] |
| RSV-positive x positive for other viruses | 1.50 |  | [0.56; 4.04] |
| Sex (male sex = 1) | 0.88 |  | [0.67; 1.15] |
| Age <2 months | 0.23 | ** | [0.16; 0.35] |
| Age 2-6 months | 0.46 | ** | [0.31; 0.68] |
| Age ≥6 months | Ref |  | - |
| Preterm | 1.10 |  | [0.77; 1.56] |
| Respiratory comorbidities | 0.88 |  | [0.49; 1.55] |
| Other comorbidities | 0.83 |  | [0.52; 1.32] |
| Constant | 6.74 |  |  |

^**^ p≤ 1%, ^*^ p≤ 5%
