# Supplementary material for: Impact of RSV test positivity, patient characteristics, and treatment characteristics on the cost of hospitalization for acute bronchiolitis in a French university medical center (2010–2015)
Source: Front Pediatr. 2023 Jul 14;11:1126229. doi: 10.3389/fped.2023.1126229 (PMC10390249; doi:10.3389/fped.2023.1126229)
Supplement: Supplementary file 5 [file Table5.docx]

**Supplementary Table 5.** Distribution of the cost and the log-transformed cost (full sample, N=1164)

|  | Cost (€) | Ln(cost) |
| --- | --- | --- |
| Mean | 4212.86 | 8.086 |
| Standard deviation | 5047.32 | 0.661 |
| Skewness | 9.27 | 0.461 |
| Kurtosis | 139.95 | 4.888 |
| 1^st^ percentile | 753.10 | 6.624 |
| 5^th^ percentile | 1190.44 | 7.082 |
| 10^th^ percentile | 1464.30 | 7.289 |
| 25^th^ percentile | 2099.81 | 7.650 |
| Median | 3248.41 | 8.086 |
| 75^th^ percentile | 4671.92 | 8.449 |
| 90^th^ percentile | 7261.31 | 8.890 |
| 95^th^ percentile | 9254.20 | 9.133 |
| 99^th^ percentile | 25808.50 | 10.158 |
