# Supplementary material for: Impact of RSV test positivity, patient characteristics, and treatment characteristics on the cost of hospitalization for acute bronchiolitis in a French university medical center (2010–2015)
Source: Front Pediatr. 2023 Jul 14;11:1126229. doi: 10.3389/fped.2023.1126229 (PMC10390249; doi:10.3389/fped.2023.1126229)
Supplement: Supplementary file 6 [file Table6.docx]

**Supplementary Table 6.** Results for the Box-Cox model, the EEE approach, and the generalized gamma model (model 2; estimation subsample n=931)

| Variables | Box-Cox | | EEE | | Generalized gamma | |
| --- | --- | --- | --- | --- | --- | --- |
|  | Coeff. | χ^2^(1) | Coeff. | Robust SE | Coeff. | Robust SE |
| RSV-positive | 0.0263^#^ | 2.82 | 0.0234 | 0.045 | 0.0657^#^ | 0.038 |
| Positive for other viruses | 0.0699^**^ | 7.91 | 0.2555^**^ | 0.088 | 0.1725^*^ | 0.070 |
| RSV-positive x positive for other viruses | 0.0747 | 1.90 | 0.0768 | 0.140 | 0.1724 | 0.135 |
| Sex (male sex = 1) | -0.0311^*^ | 4.90 | -0.0925^*^ | 0.040 | -0.0782^*^ | 0.031 |
| Age <2 months | 0.0981^**^ | 22.47 | 0.2679^**^ | 0.055 | 0.2113^**^ | 0.045 |
| Age 2-6 months | 0.0230 | 1.70 | 0.0889^#^ | 0.052 | 0.0335 | 0.041 |
| Age ≥6 months | Ref | - | Ref | - | Ref | - |
| Preterm | 0.0212 | 1.38 | 0.0782 | 0.050 | 0.0396 | 0.042 |
| Respiratory comorbidities | 0.0800^**^ | 7.89 | 0.1959^**^ | 0.076 | 0.1798^**^ | 0.070 |
| Other comorbidities | 0.1457^**^ | 39.08 | 0.4639^**^ | 0.078 | 0.3217^**^ | 0.068 |
| Pulmonary superinfections | 0.0786^**^ | 15.57 | 0.1714^**^ | 0.047 | 0.1549^**^ | 0.046 |
| Other superinfections | 0.1116^**^ | 19.84 | 0.3370^**^ | 0.073 | 0.2518^**^ | 0.071 |
| Oxygen therapy | 0.1914^**^ | 121.77 | 0.4597^**^ | 0.062 | 0.4563^**^ | 0.040 |
| X-ray imaging | 0.0723^**^ | 18.28 | 0.1952^**^ | 0.053 | 0.1420^**^ | 0.037 |
| Respiratory support | 0.2290^**^ | 65.01 | 0.6110^**^ | 0.079 | 0.5520^**^ | 0.069 |
| PICU admission | 0.0480 | 2.40 | 0.0533 | 0.096 | 0.1352^#^ | 0.076 |
| Constant | 5.0486^**^ |  | -0.9774^**a^ | 0.086 | 7.3523^**^ | 0.063 |

| Parameters | Coeff. | Robust SE | Coeff. | Robust SE | Coeff. | Robust SE |
| --- | --- | --- | --- | --- | --- | --- |
| θ | -0.1088^**^ | 0.0180 |  |  |  |  |
|  |  |  |  |  |  |  |
| λ |  |  | -0.3834^*^ | 0.169 |  |  |
| θ_1_ |  |  | 0.3361^**^ | 0.028 |  |  |
| θ_2_ |  |  | 2.5757^**^ | 0.241 |  |  |
|  |  |  |  |  |  |  |
| Ln(σ) |  |  |  |  | het^b^ | - |
| κ |  |  |  |  | -0.0624 | 0.080 |

^**^ p≤ 1%, ^*^ p≤ 5%, ^#^ p≤ 10%

^a^ To force convergence, the dependent variable was scaled by dividing by its mean

^b^ Ln(σ) was specified as a linear function of all the independent variables

EEE, Extended Estimating Equations estimator
