# Supplementary material for: Impact of RSV test positivity, patient characteristics, and treatment characteristics on the cost of hospitalization for acute bronchiolitis in a French university medical center (2010–2015)
Source: Front Pediatr. 2023 Jul 14;11:1126229. doi: 10.3389/fped.2023.1126229 (PMC10390249; doi:10.3389/fped.2023.1126229)
Supplement: Supplementary file 7 [file Table7.docx]

**Supplementary Table 7.** Comparison of models - specification tests

| Models | Breusch-Pagan heteroskedasticity test  χ^2^(1) | Pregibon’s link test  t | Pearson ρ test | Hosmer-Lemeshow test  F(10, 921) | Copas overfitting test |
| --- | --- | --- | --- | --- | --- |
| OLS – Log | 12.26  (p=0.0005) | 1.09  (p=0.2776) | ρ = 0.14  (p=0.0000) | 1.54  (p=0.1188) | 0.12  (p=0.8844) |
| Box-Cox (power = -0.109) | 0.04  (p=0.8407) | 0.37  (p=0.7080) | ρ = 0.15  (p=0.0000) | 2.03  (p=0.0273) | 0.08  (p=0.9233) |
| EEE | - | - | ρ = -0.15  (p=0.0000) | 0.63  (p=0.7891)  U-shaped | 0.32  (p=0.7299) |
| GLM (link = power(-0.5), family = gamma) | - | 0.21  (p=.8330) | ρ = -0.18  (p=0.0000) | 0.61  (p=0.8084)  U-shaped | 0.25  (p=0.7800) |
| GLM (link = power(-0.5), family = inverse Gaussian) | - | 0.84  (p=0.4010) | ρ = -0.57  (p=0.0000) | 0.58  (p=0.8313)  U-shaped | 3.91  (p=0.0213) |
| GLM (link = log, family = gamma) | - | 1.44  (p=0.1510) | ρ = 0.10  (p=0.0024) | 0.91  (p=0.5243) | 0.13  (p=0.8788) |
| GLM (link = log, family = inverse Gaussian) | - | 0.23  (p=0.8170) | ρ = 0.11  (p=0.0012) | 0.69  (p=0.7323) | 0.08  (p=0.9222) |
| Lognormal model (heteroscedastic) | - | 1.32  (p=0.1870) | ρ = 0.09  (p=0.0087) | 0.71  (p=0.7173) | 0.34  (p=0.7120) |
| Conditional density estimator (ORL) | - | - | ρ = 0.16  (p=0.0000) | 4.61  (p=0.0000) | 1.24  (p=0.2899) |
| Conditional density estimator (MNL) | - | - | ρ = 0.17  (p=0.0000) | 3.41  (p=0.0002) | 2.70  (p=0.0695) |

OLS, ordinary least squares; EEE, Extended Estimating Equations estimator; GLM, generalized linear model; ORL, ordered logit; MNL, multinomial logit
