# Supplementary material for: Impact of RSV test positivity, patient characteristics, and treatment characteristics on the cost of hospitalization for acute bronchiolitis in a French university medical center (2010–2015)
Source: Front Pediatr. 2023 Jul 14;11:1126229. doi: 10.3389/fped.2023.1126229 (PMC10390249; doi:10.3389/fped.2023.1126229)
Supplement: Supplementary file 8 [file Table8.docx]

**Supplementary Table 8.** Comparison of models - goodness-of-fit criteria

| Models | Estimation subsample (N=931) | | | | Forecast subsample (N=233) | | |
| --- | --- | --- | --- | --- | --- | --- | --- |
|  | AIC | BIC | MAPE | RMSE | MPE | MAPE | RMSE |
| OLS – Log | 16427.79^a^ | 16505.17^a^ | 1863.08 | 4452.55 | -64.34 | 1713.75 | 2886.53 |
| OLS – Box-Cox (power = -0.109) | 16414.53 | 16419.36 | 1849.01 | 4461.89 | -28.91 | 1697.10 | 2895.01 |
| EEE | - | - | 1907.58 | 4606.92 | -77.56 | 1701.65 | 2809.16 |
| GLM (link = power(-0.5), family = gamma) | 17186.20 | 17263.58 | 1895.98 | 4549.17 | -53.38 | 1689.49 | 2807.31 |
| GLM (link = power(-0.5), family = inverse Gaussian) | 24311.82 | 24389.20 | 2022.25 | 6001.71 | -124.87 | 1759.53 | 2930.34 |
| GLM (link = log, family = gamma) | 17189.34 | 17266.72 | 1889.28 | 4391.56 | -82.57 | 1741.14 | 2837.53 |
| GLM (link = log, family = inverse Gaussian) | 24311.82 | 24389.20 | 1881.13 | 4479.16 | -28.18 | 1715.71 | 2835.33 |
| Lognormal model (heteroscedastic) | 16369.94^a^ | 16524.70^a^ | 1864.50 | 4508.53 | 10.41 | 1671.39 | 2755.97 |
| Conditional density estimator (ORL) | - | - | 1903.70 | 4610.58 | -154.96 | 1742.18 | 2967.42 |
| Conditional density estimator (MNL) | - | - | 1888.75 | 4578.82 | -271.46 | 1834.96 | 2907.62 |

^a^: Initial log-likelihood + 2 x Σ ln C_i_ to allow comparison across models (Σ ln C_i_ = 7507.68)

AIC, Akaike information criterion; BIC, Bayesian information criterion; MAPE, mean absolute prediction error; MPE, mean prediction error; RMSE, root mean square error; OLS, ordinary least squares; EGLM, extended generalized linear model; GLM, generalized linear model; ORL, ordered logit; MNL, multinomial log.
